# Synthesis without meta-analysis (SWiM) in systematic reviews: reporting guideline

**DOI:** 10.1136/bmj.l6890

**Published:** 2020-01-16

**Authors:** Mhairi Campbell, Joanne E McKenzie, Amanda Sowden, Srinivasa Vittal Katikireddi, Sue E Brennan, Simon Ellis, Jamie Hartmann-Boyce, Rebecca Ryan, Sasha Shepperd, James Thomas, Vivian Welch, Hilary Thomson

**Affiliations:** 1 MRC/CSO Social and Public Health Sciences Unit, University of Glasgow, UK; 2 School of Public Health and Preventive Medicine, Monash University, Melbourne, Australia; 3 Centre for Reviews and Dissemination, University of York, York, UK; 4 Centre for Guidelines, National Institute for Health and Care Excellence, London, UK; 5 Nuffield Department of Primary Care Health Sciences, University of Oxford, Oxford, UK; 6 School of Psychology and Public Health, La Trobe University, Melbourne, Australia; 7 Nuffield Department of Population Health, University of Oxford, Oxford, UK; 8 Evidence for Policy and Practice Information and Coordinating Centre, University College London, London, UK; 9 Bruyere Research Institute, Ottawa, Canada

## Abstract

In systematic reviews that lack data amenable to meta-analysis, alternative synthesis methods are commonly used, but these methods are rarely reported. This lack of transparency in the methods can cast doubt on the validity of the review findings. The Synthesis Without Meta-analysis (SWiM) guideline has been developed to guide clear reporting in reviews of interventions in which alternative synthesis methods to meta-analysis of effect estimates are used. This article describes the development of the SWiM guideline for the synthesis of quantitative data of intervention effects and presents the nine SWiM reporting items with accompanying explanations and examples.

**Summary points**

- Systematic reviews of health related interventions often use alternative methods of synthesis to meta-analysis of effect estimates, methods often described as “narrative synthesis”
- Serious shortcomings in reviews that use “narrative synthesis” have been identified, including a lack of description of the methods used; unclear links between the included data, the synthesis, and the conclusions; and inadequate reporting of the limitations of the synthesis
- The Synthesis Without Meta-analysis (SWiM) guideline is a nine item checklist to promote transparent reporting for reviews of interventions that use alternative synthesis methods
- The SWiM items prompt users to report how studies are grouped, the standardised metric used for the synthesis, the synthesis method, how data are presented, a summary of the synthesis findings, and limitations of the synthesis
- The SWiM guideline has been developed using a best practice approach, involving extensive consultation and formal consensus

Decision makers consider systematic reviews to be an essential source of evidence.^1^ Complete and transparent reporting of the methods and results of reviews allows users to assess the validity of review findings. The Preferred Reporting Items for Systematic Reviews and Meta-Analyses (PRISMA; http://www.prisma-statement.org/) statement, consisting of a 27 item checklist, was developed to facilitate improved reporting of systematic reviews.^2^ Extensions are available for different approaches to conducting reviews (for example, scoping reviews^3^), reviews with a particular focus (for example, harms^4^), and reviews that use specific methods (for example, network meta-analysis.^5^) However, PRISMA provides limited guidance on reporting certain aspects of the review, such as the methods for presentation and synthesis, and no reporting guideline exists for synthesis without meta-analysis of effect estimates. We estimate that 32% of health related systematic reviews of interventions do not do meta-analysis,^6^ ^7^ ^8^ instead using alternative approaches to synthesis that typically rely on textual description of effects and are often referred to as narrative synthesis.^9^ Recent work highlights serious shortcomings in the reporting of narrative synthesis, including a lack of description of the methods used, lack of transparent links between study level data and the text reporting the synthesis and its conclusions, and inadequate reporting of the limitations of the synthesis.^7^ This suggests widespread lack of familiarity and misunderstanding around the requirements for transparent reporting of synthesis when meta-analysis is not used and indicates the need for a reporting guideline.

## Scope of SWiM reporting guideline

This paper presents the Synthesis Without Meta-analysis (SWiM) reporting guideline. The SWiM guideline is intended for use in systematic reviews examining the quantitative effects of interventions for which meta-analysis of effect estimates is not possible, or not appropriate, for a least some outcomes.^10^ Such situations may arise when effect estimates are incompletely reported or because characteristics of studies (such as study designs, intervention types, or outcomes) are too diverse to yield a meaningful summary estimate of effect.^11^ In these reviews, alternative presentation and synthesis methods may be adopted, (for example, calculating summary statistics of intervention effect estimates, vote counting based on direction of effect, and combining P values), and SWiM provides guidance for reporting these methods and results.^11^ Specifically, the SWiM guideline expands guidance on “synthesis of results” items currently available, such as PRISMA (items 14 and 21) and RAMESES (items 11, 14, and 15).^2^ ^12^ ^13^ SWiM covers reporting of the key features of synthesis including how studies are grouped, synthesis methods used, presentation of data and summary text, and limitations of the synthesis.

SWiM is not intended for use in reviews that synthesise qualitative data, for which reporting guidelines are already available, including ENTREQ for qualitative evidence synthesis and eMERGe for meta-ethnography.^14^ ^15^

## Development of SWiM reporting guideline

A protocol for the project is available,^10^ and the guideline development was registered with the EQUATOR Network, after confirmation that no similar guideline was in development. All of the SWiM project team are experienced systematic reviewers, and one was a co-author on guidance on the conduct of narrative synthesis (AS).^9^ A project advisory group was convened to provide greater diversity in expertise. The project advisory group included representatives from collaborating Cochrane review groups, the Campbell Collaboration, and the UK National Institute for Health and Care Excellence (see supplementary file 1).

The project was informed by recommendations for developing guidelines for reporting of health research.^16^ We assessed current practice in reporting synthesis of effect estimates without meta-analysis and used the findings to devise an initial checklist of reporting items in consultation with the project advisory group. We invited 91 people, all systematic review methodologists or authors of reviews that synthesised results from studies without using meta-analysis, to participate in a three round Delphi exercise, with a response rate of 48% (n=44/91) in round one, 54% (n=37/68) in round two, and 82% (n=32/39) in round three. The results were discussed at a consensus meeting of an expert panel (the project advisory group plus one additional methodological expert) (see supplementary file 1). After the meeting, we piloted the revised guideline to assess ease of use and face validity. Eight systematic reviewers with varying levels of experience, who had not been involved in the Delphi exercise, were asked to read and apply the guideline. We conducted short interviews with the pilot participants to identify any clarification needed in the items or their explanations. We subsequently revised the items and circulated them for comment among the expert panel, before finalising them. Full methodological details of the SWiM guideline development process are provided in supplementary file 1.

## Synthesis without meta-analysis reporting items

We identified nine items to guide the reporting of synthesis without meta-analysis. Table 1 shows these SWiM reporting items. An online version is available at www.equator-network.org/reporting-guidelines. An explanation and elaboration for each of the reporting items is provided below. Examples to illustrate the reporting items and explanations are provided in supplementary file 2.

**Table 1:** Synthesis Without Meta-analysis (SWiM) items: SWiM is intended to complement and be used as an extension to PRISMA

SWiM reporting item	Item description	Page in manuscript where item is reported	Other*
**Methods**			
1 Grouping studies for synthesis	1a) Provide a description of, and rationale for, the groups used in the synthesis (eg, groupings of populations, interventions, outcomes, study design)		
	1b) Detail and provide rationale for any changes made subsequent to the protocol in the groups used in the synthesis		
2 Describe the standardised metric and transformation methods used	Describe the standardised metric for each outcome. Explain why the metric(s) was chosen and describe any methods used to transform the intervention effects, as reported in the study, to the standardised metric, citing any methodological guidance consulted		
3 Describe the synthesis methods	Describe and justify the methods used to synthesise the effects for each outcome when it was not possible to undertake a meta-analysis of effect estimates		
4 Criteria used to prioritise results for summary and synthesis	Where applicable, provide the criteria used, with supporting justification, to select the particular studies, or a particular study, for the main synthesis or to draw conclusions from the synthesis (eg, based on study design, risk of bias assessments, directness in relation to the review question)		
5 Investigation of heterogeneity in reported effects	State the method(s) used to examine heterogeneity in reported effects when it was not possible to undertake a meta-analysis of effect estimates and its extensions to investigate heterogeneity		
6 Certainty of evidence	Describe the methods used to assess the certainty of the synthesis findings		
7 Data presentation methods	Describe the graphical and tabular methods used to present the effects (eg, tables, forest plots, harvest plots)		
	Specify key study characteristics (eg, study design, risk of bias) used to order the studies, in the text and any tables or graphs, clearly referencing the studies included		
**Results**			
8 Reporting results	For each comparison and outcome, provide a description of the synthesised findings and the certainty of the findings. Describe the result in language that is consistent with the question the synthesis addresses, and indicate which studies contribute to the synthesis		
**Discussion**			
9 Limitations of the synthesis	Report the limitations of the synthesis methods used and/or the groupings used in the synthesis and how these affect the conclusions that can be drawn in relation to the original review question		

PRISMA=Preferred Reporting Items for Systematic Reviews and Meta-Analyses.

* If the information is not provided in the systematic review, give details of where this information is available (eg, protocol, other published papers (provide citation details), or website (provide the URL)).

### Item 1: grouping studies for synthesis

#### 1a) Description

Provide a description of, and rationale for, the groups used in the synthesis (for example, groupings of interventions, population, outcomes, study design).

#### 1a) Explanation

Methodological and clinical or conceptual diversity may occur (for example, owing to inclusion of diverse study designs, outcomes, interventions, contexts, populations), and it is necessary to clearly report how these study characteristics are grouped for the synthesis, along with the rationale for the groups (see Cochrane Handbook Chapter 3^17^). Although reporting the grouping of study characteristics in all reviews is important, it is particularly important in reviews without meta-analysis, as the groupings may be less evident than when meta-analysis is used.

Providing the rationale, or theory of change, for how the intervention is expected to work and affect the outcome(s) will inform authors’ and review users’ decisions about the appropriateness and usefulness of the groupings. A diagram, or logic model,^18^ ^19^ can be used to visually articulate the underlying theory of change used in the review. If the theory of change for the intervention is provided in full elsewhere (for example, in the protocol), this should be referenced. In Cochrane reviews, the rationale for the groups can be outlined in the section “How the intervention is expected to work.”

#### 1b) Description

Detail and provide rationale for any changes made subsequent to the protocol in the groups used in the synthesis.

#### 1b) Explanation

Decisions about the planned groups for the syntheses may need to be changed following study selection and data extraction. This may occur as a result of important variations in the population, intervention, comparison, and/or outcomes identified after the data are collected, or where limited data are available for the pre-specified groupings, and the groupings may need to be modified to facilitate synthesis (Cochrane Handbook Chapter 2^20^). Reporting changes to the planned groups, and the reason(s) for these, is important for transparency, as this allows readers to assess whether the changes may have been influenced by study findings. Furthermore, grouping at a broader level of (any or multiple) intervention, population, or outcome will have implications for the interpretation of the synthesis findings (see item 8).

### Item 2: describe the standardised metric and transformation method used

#### Description

Describe the standardised metric for each outcome. Explain why the metric(s) was chosen, and describe any methods used to transform the intervention effects, as reported in the study, to the standardised metric, citing any methodological guidance used.

#### Explanation

The term “standardised metric” refers to the metric that is used to present intervention effects across the studies for the purpose of synthesis or interpretation, or both. Examples of standardised metrics include measures of intervention effect (for example, risk ratios, odds ratios, risk differences, mean differences, standardised mean differences, ratio of means), direction of effect, or P values. An example of a statistical method to convert an odds ratio to a standardised mean difference is that proposed by Chinn (2000).^21^ For other methods and metrics, see Cochrane Handbook Chapter 6.^22^

### Item 3: describe the synthesis methods

#### Description

Describe and justify the methods used to synthesise the effects for each outcome when it was not possible to undertake a meta-analysis of effect estimates.

#### Explanation

For various reasons, it may not be possible to do a meta-analysis of effect estimates. In these circumstances, other synthesis methods need to be considered and specified. Examples include combining P values, calculating summary statistics of intervention effect estimates (for example, median, interquartile range) or vote counting based on direction of effect. See table 2 for a summary of possible synthesis methods (for further details, see McKenzie and Brennan 2019^11^). Justification should be provided for the chosen synthesis method.

**Table 2:** Questions answered according to types of synthesis methods and types of data used

Question answered	Synthesis method	Minimum data required			
		Estimate of effect	Variance	Direction of effect	Precise P value
What is the common intervention effect? What is the average intervention effect? Which intervention, of multiple, is most effective? What factors modify the magnitude of the intervention effects?	Meta-analysis of effect estimates and extensions (eg, sub-group analysis, meta-regression, network meta-analysis)	✔	✔	-	-
What is the range and distribution of observed effects?	Summarising effect estimates	✔	-	-	-
Is there evidence of an effect in at least one study?	Combining P values	-	-	✔	✔
Is there any evidence of an effect?	Vote counting based on direction of effect	-	-	✔	-

Abbreviated from table 12.2.a of McKenzie and Brennan 2019.^11^

### Item 4: criteria used to prioritise results for summary and synthesis

#### Description

Where applicable, provide the criteria used, with supporting justification, to select particular studies, or a particular study, for the main synthesis or to draw conclusions from the synthesis (for example, based on study design, risk of bias assessments, directness in relation to the review question).

#### Explanation

Criteria may be used to prioritise the reporting of some study findings over others or to restrict the synthesis to a subset of studies. Examples of criteria include the type of study design (for example, only randomised trials), risk of bias assessment (for example, only studies at a low risk of bias), sample size, the relevance of the evidence (outcome, population/context, or intervention) pertaining to the review question, or the certainty of the evidence. Pre-specification of these criteria provides transparency as to why certain studies are prioritised and limits the risk of selective reporting of study findings.

### Item 5: investigation of heterogeneity in reported effects

#### Description

State the method(s) used to examine heterogeneity in reported effects when it is not possible to do a meta-analysis of effect estimates and its extensions to investigate heterogeneity.

#### Explanation

Informal methods to investigate heterogeneity in the findings may be considered when a formal statistical investigation using methods such as subgroup analysis and meta-regression is not possible. Informal methods could involve ordering tables or structuring figures by hypothesised modifiers such as methodological characteristics (for example, study design), subpopulations (for example, sex, age), intervention components, and/or contextual/setting factors (see Cochrane Handbook Chapter 12^11^). The methods used and justification for the chosen methods should be reported. Investigations of heterogeneity should be limited, as they are rarely definitive; this is more likely to be the case when informal methods are used. It should also be noted if the investigation of heterogeneity was not pre-specified.

### Item 6: certainty of evidence

#### Description

Describe the methods used to assess the certainty of the synthesis findings.

#### Explanation

The assessment of the certainty of the evidence should aim to take into consideration the precision of the synthesis finding (confidence interval if available), the number of studies and participants, the consistency of effects across studies, the risk of bias of the studies, how directly the included studies address the planned question (directness), and the risk of publication bias. GRADE (Grading of Recommendations, Assessment, Development and Evaluations) is the most widely used framework for assessing certainty (Cochrane Handbook Chapter 14^23^). However, depending on the synthesis method used, assessing some domains (for example, consistency of effects when vote counting is undertaken) may be difficult.

### Item 7: data presentation methods

#### Description

Describe the graphical and tabular methods used to present the effects (for example, tables, forest plots, harvest plots).

Specify key study characteristics (for example, study design, risk of bias) used to order the studies, in the text and any tables or graphs, clearly referencing the studies included

#### Explanation

Study findings presented in tables or graphs should be ordered in the same way as the syntheses are reported in the narrative text to facilitate the comparison of findings from each included study. Key characteristics, such as study design, sample size, and risk of bias, which may affect interpretation of the data, should also be presented. Examples of visual displays include forest plots,^24^ harvest plots,^25^ effect direction plots,^26^ albatross plots,^27^ bubble plots,^28^ and box and whisker plots.^29^ McKenzie and Brennan (2019) provide a description of these plots, when they should be used, and their pros and cons.^11^

### Item 8: reporting results

#### Description

For each comparison and outcome, provide a description of the synthesised findings and the certainty of the findings. Describe the result in language that is consistent with the question the synthesis addresses and indicate which studies contribute to the synthesis.

#### Explanation

For each comparison and outcome, a description of the synthesis findings should be provided, making clear which studies contribute to each synthesis (for example, listing in the text or tabulated). In describing these findings, authors should be clear about the nature of the question(s) addressed (see table 2, column 1), the metric and synthesis method used, the number of studies and participants, and the key characteristics of the included studies (population/settings, interventions, outcomes). When possible, the synthesis finding should be accompanied by a confidence interval.An assessment of the certainty of the effect should be reported.

Results of any investigation of heterogeneity should be described, noting if it was not pre-planned and avoiding over-interpretation of the findings.

If a pre-specified logic model was used, authors may report any changes made to the logic model during the review or as a result of the review findings.^30^

### Item 9: limitations of the synthesis

#### Description

Report the limitations of the synthesis methods used and/or the groupings used in the synthesis and how these affect the conclusions that can be drawn in relation to the original review question.

#### Explanation

When reporting limitations of the synthesis, factors to consider are the standardised metric(s) used, the synthesis method used, and any reconfiguration of the groups used to structure the synthesis (comparison, intervention, population, outcome).

The choice of metric and synthesis method will affect the question addressed (see table 2). For example, if the standardised metric is direction of effect, and vote counting is used, the question will ask “is there any evidence of an effect?” rather than “what is the average intervention effect?” had a random effects meta-analysis been used.

Limitations of the synthesis might arise from post-protocol changes in how the synthesis was structured and the synthesis method selected. These changes may occur because of limited evidence, or incompletely reported outcome or effect estimates, or if different effect measures are used across the included studies. These limitations may affect the ability of the synthesis to answer the planned review question—for example, when a meta-analysis of effect estimates was planned but was not possible.

## Discussion

The SWiM reporting guideline is intended to facilitate transparent reporting of the synthesis of effect estimates when meta-analysis is not used. The guideline relates specifically to transparently reporting synthesis and presentation methods and results, and it is likely to be of greatest relevance to reviews that incorporate diverse sources of data that are not amenable to meta-analysis. The SWiM guideline should be used in conjunction with other reporting guidelines that cover other aspects of the conduct of reviews, such as PRISMA.^31^ We intend SWiM to be a resource for authors of reviews and to support journal editors and readers in assessing the conduct of a review and the validity of its findings.

The SWiM reporting items are intended to cover aspects of presentation and synthesis of study findings that are often left unreported when methods other than meta-analysis have been used.^7^ These include reporting of the synthesis structure and comparison groupings (items 1, 4, 5, and 6), the standardised metric used for the synthesis (item 2), the synthesis method (items 3 and 9), presentation of data (item 7), and a summary of the synthesis findings that is clearly linked to supporting data (item 8). Although the SWiM items have been developed specifically for the many reviews that do not include meta-analysis, SWiM promotes the core principles needed for transparent reporting of all synthesis methods including meta-analysis. Therefore, the SWiM items are relevant when reporting synthesis of quantitative effect data regardless of the method used.

Reporting guidelines are sometimes interpreted as providing guidance on conduct or used to assess the quality of a study or review; this is not an appropriate application of a reporting guideline, and SWiM should not be used to guide the conduct of the synthesis. For guidance on how to conduct synthesis using the methods referred to in SWiM, we direct readers to the second edition of the Cochrane Handbook for Systematic Reviews of Interventions, specifically chapter 12.^11^ Although an overlap inevitably exists between reporting and conduct, the SWiM reporting guideline is not intended to be prescriptive about choice of methods, and the level of detail for each item should be appropriate. For example, investigation of heterogeneity (item 5) may not always be necessary or useful. In relation to SWiM, we anticipate that the forthcoming update of PRISMA will include new items covering a broader range of synthesis methods,^32^ but it will not provide detailed guidance and examples on synthesis without meta-analysis.

The SWiM reporting guideline emerged from a project aiming to improve the transparency and conduct of narrative synthesis (ICONS-Quant: Improving the CONduct and reporting of Narrative Synthesis).^10^ Avoidance of the term “narrative synthesis” in SWiM is a deliberate move to promote clarity in the methods used in reviews in which the synthesis does not rely on meta-analysis. The use of narrative is ubiquitous across all research and can serve a valuable purpose in the development of a coherent story from diverse data.^33^ ^34^ However, within the field of evidence synthesis, narrative approaches to synthesis of quantitative effect estimates are characterised by a lack of transparency, making assessment of the validity of their findings difficult.^7^ Together with the recently published guidance on conduct of alternative methods of synthesis,^11^ the SWiM guideline aims to improve the transparency of, and subsequently trust in, the many reviews that synthesise quantitative data without meta-analysis, particularly for reviews of intervention effects.

## Notes

Contributors: All authors contributed to the development of SWiM. HT had the idea for the study. HT, SVK, AS, JEM, and MC designed the study methods. JT, JHB, RR, SB, SE, SS, and VW contributed to the consensus meeting and finalising the guideline items. MC prepared the first draft of the manuscript, and all authors critically reviewed and approved the final manuscript. The corresponding author attests that all listed authors meet authorship criteria and that no others meeting the criteria have been omitted. HT is the guarantor.

Project advisory group members: Simon Ellis, Jamie Hartmann-Boyce, Mark Petticrew, Rebecca Ryan, Sasha Shepperd, James Thomas, Vivian Welch.

Expert panel members: Sue Brennan, Simon Ellis, Jamie Hartmann-Boyce, Rebecca Ryan, Sasha Shepperd, James Thomas, Vivian Welch.

Ethical approval: Ethical approval was obtained from the University of Glasgow College of Social Sciences Ethics Committee (reference number 400170060).

*Transparency: The lead author affirms that the manuscript is an honest, accurate, and transparent account of the study being reported; that no important aspects of the study have been omitted; and that any discrepancies from the study as planned (and, if relevant, registered) have been explained.*

Patient and public involvement: This research was done without patient involvement. Patients were not invited to comment on the study design and were not consulted to develop outcomes or interpret the results.

Dissemination to participants and related patient and public communities: The authors plan to disseminate the research through peer reviewed publications, national and international conferences, webinars, and an online training module and by establishing an email discussion group.

## Web extra

Extra material supplied by authors

**Web appendix:** Supplementary file 1

**Web appendix:** Supplementary file 2
