# Supplementary material for: Synthesis without meta-analysis (SWiM) in systematic reviews: reporting guideline
Source: BMJ. 2020 Jan 16;368:l6890. doi: 10.1136/bmj.l6890 (PMC7190266; doi:10.1136/bmj.l6890)
Supplement: Supplementary file 1 — Web appendix: Supplementary file 1 [file camm051291.ww1.pdf]

Synthesis without meta-analysis (SWiM) in systematic reviews: reporting guideline

Supplementary file 1

Methodology for developing Synthesis Without Meta-analysis reporting guideline

This supplementary file outlines the methods used to develop the Synthesis Without Meta-analysis (SWiM) reporting items. There are five member of the SWiM team, all with experience of conducting systematic reviews. AS is an author of the published guidance on narrative synthesis funded by the UK's Economic and Social Research Council.[1] HT and SVK are Cochrane editors, and JEM is a co-convenor of Cochrane Statistical Methods Group. Recommendations for developing health research guidelines[2] were used to steer the development of this reporting guideline. We convened a project advisory group to provide expert advice and governance for the project. Members of the advisory group were recruited from the collaborating Cochrane Review Groups (Effective Practice and Organisation of Care, Tobacco Addiction, and Consumers and Communication), representatives from the Campbell Collaboration Methods Group and the National Institute for Health and Care Excellence (NICE), and experts in narrative synthesis methodologies. A protocol for the project is available.[3] While the project is called ICONS-Quant (Improving the CONduct and reporting of Narrative Synthesis), the reporting guideline is labelled SWiM to differentiate it from "narrative synthesis" in general which may involve other methods or include synthesis of qualitative data. The focus of this guideline is on synthesis of quantitative effects when meta-analysis is not possible.

1. **Assessing currently available guidance for reporting synthesis without meta-analysis**

We conducted a purposive search of articles and textbooks for key texts on conducting narrative synthesis of quantitative data.[4] Through this we established that the work by Popay et al[1] provides the most complete guidance on conducting narrative synthesis, including both quantitative and qualitative data synthesis. Some more limited guidance was provided by textbooks and reports of systematic review methods.[5-9] It became apparent as this project progressed, that the term "narrative synthesis" is used to refer to various methods of synthesis. Therefore, to clarify that this reporting guideline has been developed for the synthesis of quantitative data in systematic reviews of interventions, the term "synthesis without meta-analysis" was adopted.

To ensure there were no guidelines published or in development for reporting narrative synthesis of quantitative data, we checked the Equator Network library of reporting guidelines (http://www. equator- network.org). The Cochrane Methodological Expectations of Cochrane Intervention Reviews (MECIR) standards for conducting and reporting Cochrane reviews provide one item relating to reporting of the data synthesis (item R51) "Describe any methods used for combining results across studies. Where data have been combined in statistical software external to RevMan, reference the software, commands and settings used to run the analysis."[10] The Preferred Reporting Items for Systematic Reviews and Meta-Analyses (PRISMA) statement provides two items that refer to reporting of the synthesis of results. The PRISMA statement currently concentrates on reporting meta-analysis, and does not provide detailed guidance on how to report alternative methods to meta-analysis. Item 14 states "Describe the methods of handling data and combining results of studies, if done, including measures of consistency (e.g., I^2) for each meta-analysis".[11 page 5] Item 21 states "Present results of each meta-analysis done, including confidence intervals and measures of consistency".[11 page 5] PRISMA for systematic review protocols (PRISMA-P) provides one sub-item prompting authors to report the planned synthesis,[12] however PRISMA-P is intended for reporting protocols rather than reviews. In the context of these existing reporting guidelines for reviews, SWiM therefore aims to expand PRISMA "synthesis of results" items 14 and 21.

2. Assessing current reporting of synthesis without meta-analysis in reviews

A sample of Cochrane reviews was examined to establish current reporting of synthesis methods when meta-analysis of effect estimates was not used. The sample comprised all reviews published between April 2016 and April 2017, excluding overviews (i.e. reviews of reviews), empty reviews (no studies included), withdrawn reviews, diagnostic test accuracy reviews, methodological reviews, or reviews that provided no data for the primary outcome (see Figure S1 for the flow chart of the screening process). For the first stage of assessment, we examined what synthesis methods were used for the primary outcome. Approximately half of the sample conducted meta-analysis (49%; n=347/714), 16% (n=113/714) used an alternative method to meta-analysis as the main method of synthesis, and 36% (n=254/714) used a combination of meta-analysis with alternative methods used for the data or studies not included in the meta-analysis. Therefore, approximately half (51%) of the sample reviews used synthesis methods alternative to meta-analysis.

From the 16% (n=113/714) of reviews that did not use meta-analysis, we selected the reviews that attempted to synthesise the data in some way (i.e. we excluded the reviews that *only* reported descriptions of individual studies with no attempt to combine). This resulted in 45 reviews. We included a further 15 reviews recommended by the project advisory group. These recommended reviews were Cochrane reviews that included synthesis without meta-analysis, published outside the sampling dates. This sample of 60 reviews was examined to find out what was, and what was not, being reported for the synthesis.

We adapted a data extraction template, guided by the ESRC guidance on narrative synthesis[1] and further texts on conducting narrative synthesis[5-8]. A version of this template was used successfully in a previous project.[4] Information was gathered on methods reported, methods used to synthesise data, methods used to examine heterogeneity of reported results, and reported limitations of the synthesis.

We found that almost half of reviews (n=28/60, 47%) did not state what method was being used as the main form of synthesis, and over three quarters (n=49/60, 82%) did not provide a description of these "narrative synthesis" methods. Methods to increase the transparency of the synthesis were frequently missing, such as providing a table that displayed characteristics of *all* the included studies (in one table) (n=40, 67% not provided), and, similarly a table that included *all* the study results (n=28/60, 47% not provided). Also, frequently missing was examination of reasons for any identified heterogeneity between study results (n=16/31, 51% not provided). Finally, around a quarter of reviews (n=16/60, 27%) did not report limitations of the synthesis methods they used. These results were used to inform the development of draft reporting items for the Delphi consultation exercise.

3. Delphi consultation exercise and consensus meeting

Delphi processes aim to gather the opinions of a broad range of experts. We conducted an online modified Delphi exercise, with Round One providing a list of draft items rather than idea gathering.[13] This is the standard approach to elicit expert opinion for the purposes of developing research reporting guidelines and previous reporting guidelines have used this modified Delphi method.[14 15] Ethical approval was obtained from the University of Glasgow College of Social Sciences Ethics Committee (reference number 400170060). Data Garden at the University of Stirling provided the online platform for the distribution and collation of the Delphi exercise.

4.1 Delphi participants

We invited 81 systematic review authors, all with experience of conducting narrative synthesis to take part in the Delphi exercise. We gained a further 10 participants from recommendations from existing participants, bringing the total number of invited participants to 91. Knowledge within the SWiM management team and project advisory group was used to compile an initial list of people with professional expertise of narrative synthesis. This list also included systematic review authors who had undertaken narrative synthesis, identified in the assessment of current reporting in Cochrane reviews. Potential participants were contacted via their publically available work email addresses and invited to participate in the Delphi exercise. The invitation outlined the purpose of the exercise and provided a link to the online Delphi exercise. The introductory page explained the process, instructions for completing the exercise, and a participant information sheet. The participant information sheet provided information about the SWiM project including: provided information would be de-identified and used anonymously; every effort would be made to maintain confidentiality; the exercise was conducted using a secure server; and the results were to be saved onto an encrypted computer and stored according to MRC policy.

The Delphi respondents (51%, n=46/91) provided basic background information about their job role, type and country of organisation, and experience conducting systematic reviews. The information was collated and summarised: 87% (n=40/46) were based at a university; 22% (n=10/46) at a non-profit organisation (some participants reported roles at multiple institutions); 24 participants were based in the UK, ten in Canada, six in Australia, three in Switzerland and two in the United States (Table S1). We invited individuals from several low and middle-income countries, however none participated; we hypothesize that the patterns of responses may reflect the countries that are the main producers of non-meta-analysis (narrative synthesis) reviews. Over three quarters of the respondents (78%, n=36/46) had conducted more than six systematic reviews, 63% (n=29/46) had conducted more than six narrative synthesis systematic reviews.

Table S1 Delphi respondents' self-reported background details

**Role***	**n (%)<br>n = 46**
Systematic reviewer	46 (100%)
Quantitative researcher	10 (22%)
Mixed methods researcher	19 (41%)
Statistician	1 (2%)
Editor (peer review journal / Cochrane)	17 (37%)
Practitioner	3 (7%)
Other: clinical practice guideline methodologist (1), information scientist (1), manager of editorial process (1), qualitative researcher (2), statistician (1), trainer (1)	7 (15%)
**Institution***	**n**
University	40 (87%)
Non-profit organisation	10 (22%)
Other:<br>HTA organization	1 (2%)
**Country of institution**	**n**
UK	24 (52%)
Canada	10 (22%)
Australia	6 (13%)
Other: Switzerland (3), USA (2)	5 (11%)

Missing	1 (2%)
**Number of systematic reviews conducted**	**n**
6+ review	36 (78%)
2-5 reviews	10 (22%)
**Number of narrative synthesis reviews**	**n**
6+ review	29 (63%)
2-5 reviews	11 (24%)
1 review	3 (7%)
0 reviews	2 (4%)
Missing	1 (2%)

*Some participants reported multiple roles/institutions

4.2 Delphi Round One

Round One presented thirteen items (five items had sub-items). The items were developed in consultation with the project advisory group, from existing guidance and using the results of the assessment of current reporting in reviews, as described above in section 2. The participants were asked to rate each item on a four point Likert scale (essential, desirable, possible, omit). The rating scale has been successfully used to develop other reporting guidelines (e.g. PRISMA for Abstracts,[15] TIDieR[14]). Participants were asked to comment on the appropriateness and wording of each item, and to give any further suggestions on what should be included in the reporting guideline.

There was a 48% (n=44/91) response rate for Round One. The project team used the rating and comments to adapt the items for Round Two. There was high agreement for inclusion of the items (Table S2).

Table S2 Summary results for Round One of Delphi exercise

**Item**	**Essential (n)**	**Desirable (n)**	**Possible (n)**	**Omit (n)**	**Essential (%)**	**Essential + Desirable (%)**
1 RATIONALE	24	20	0	2	52.2	**95.7**
2 OBJECTIVES OF NS	40	4	0	1	88.9	**97.8**
3 JUSTIFICATION FOR NS	39	4	0	2	86.7	**95.6**
4 METHODOLOGICAL GUIDANCE	27	12	4	2	60.0	**86.7**
5 OUTCOMES	31	6	3	4	70.5	**84.1**
6 MANAGEMENT OF HETEROGENEITY IN STUDY CHARACTERISTICS						
6a (how data categorized)	40	5	0	0	88.9	**100.0**
6b (rationale for categorization)	27	14	2	1	61.4	**93.2**
6c (when categorization occurred)	14	21	8	0	31.8	**81.4**
7 TRANSFORMING DATA INTO COMMON METRIC						
7a (specify common metric)	22	14	6	3	48.9	**80.0**
7b (how metric calculated)	19	16	5	4	43.2	**79.5**
7c (how metric combined)	21	13	6	4	47.7	**77.3**
8 WEIGHTING	19	19	5	1	43.2	**86.4**
9 INVESTIGATION OF HETEROGENEITY IN REPORTED EFFECTS						
9a (which outcomes assessed)	19	17	6	2	43.2	**81.8**
9b (how heterogeneity reported)	16	23	3	2	36.4	**88.6**

9c (was heterogeneity explained)	13	23	4	4	29.6	**81.8**
10 INCLUSION OF STUDIES	41	1	2	0	93.2	**95.5**
11 DATA PRESENTATION METHODS						
11a (methods used)	22	14	6	1	51.2	**83.7**
11b (order in tables and text)	13	20	8	2	30.2	**76.7**
12 TEXT REPORTING NARRATIVE SYNTHESIS OF SUMMARISED DATA						
12a (studies in each comparison)	38	3	1	2	86.6	**93.2**
12b (summary for each comparison)	39	5	0	0	100.0	**100.0**
13 LIMITATIONS OF THE SYNTHESIS	38	5	1	0	86.6	**97.7**

4.3 Delphi Round Two

Feedback from the comments and ratings in Round One indicated that three items should be incorporated into relevant pre-existing items for Round Two. "Objectives of NS" was incorporated into "Reporting results", "Methodological guidance" was incorporated into "Common metric", and "Outcomes" was incorporated into "Selection of studies". Many invitees in the UK were unable to participate in Round One due to industrial action and indicated that they would be interested in participating in Round Two.

For Round Two, there was a 54% (n=37 of 68) response rate. The revised items were circulated to the Delphi participants in Round Two for further rating and comments. There were ten items in Round Two, two with sub-items. All of the items were rated "Essential" or "Desirable" by over 80% of the participants (Table S3). Again, in response to comments and ratings, the items were revised.

Table S3 Summary results for Round Two of Delphi exercise

**Item**	**Essential (n)**	**Desirable (n)**	**Possible (n)**	**Omit (n)**	**Essential (%)**	**Essential + Desirable (%)**
1 RATIONALE	22	14	2	0	57.9	**94.7**
2 JUSTIFICATION FOR NS	33	3	0	1	89.2	**97.3**
3 MANAGEMENT OF DIVERSITY IN STUDY CHARACTERISTICS						
3a (rationale for groups used)	34	2	1	0	91.9	**97.3**
3b (changes to groups with rationale)	14	20	1	3	36.8	**89.5**
4 TRANSFORMING EFFECTS INTO COMMON METRIC						
4a (statistical methods used to create metric)	23	9	2	3	62.2	**86.5**
4b (visual methods used to create metric)	16	14	3	4	43.2	**81.1**
4c (if multiple metrics, which metrics applied to which outcomes)	31	1	3	3	81.6	**84.2**
5 STATISTICAL SYNTHESIS METHODS	24	9	2	2	64.9	**89.2**
6 SELECTION OF STUDIES	23	12	0	2	62.2	**94.6**
7 INVESTIGATION OF HETEROGENEITY IN REPORTED EFFECTS	15	22	0	0	40.5	**100.0**
8 DATA PRESENTATION METHODS	25	9	2	0	69.4	**94.4**
9 REPORTING RESULTS	35	1	1	0	94.6	**97.3**

10 LIMITATIONS OF THE SYNTHESIS	35	1	1	0	94.6	**97.3**

4.4 Delphi Round Three

In the third round of the Delphi exercise, there were ten items (two with sub-items). Analysis from Round Two led to the sub-items for "Transforming data into a common metric" being combined into one item. The item labelled "Managing diversity in study characteristics" was split into two sub-items. The revised items were circulated for final rating and comments. There was an 82% (n=32/39) response rate. Two individuals who did not complete Round Two contacted the project to request they be included in Round Three. The items were rated as either "Essential" or "Desirable" by over 90% of the participants (Table S4).

Table S4 Summary results for Round Three of Delphi exercise

Item	Item label	Essential (n)	Desirable (n)	Possible (n)	Omit (n)	Essential (%)	Essential + Desirable (%)
1 RATIONALE		19	11	0	1	61.3	**96.8**
2 JUSTIFICATION FOR NS		30	0	0	1	96.8	**96.8**
3 MANAGEMENT OF DIVERSITY IN STUDY CHARACTERISTICS							
3a (rationale for groups used)		29	1	1	1	90.6	**93.8**
3b ((changes to groups with rationale)		11	19	0	1	35.5	**96.8**
4 TRANSFORMING EFFECTS INTO COMMON METRIC		27	2	2	1	84.4	**90.6**
5 STATISTICAL SYNTHESIS METHODS		24	5	1	1	77.4	**93.5**
6 SELECTION OF STUDIES		30	1	0	1	93.8	**96.9**
7 INVESTIGATION OF HETEROGENEITY IN REPORTED EFFECTS		12	18	1	0	38.7	**96.8**
8 DATA PRESENTATION METHODS		28	1	1	1	90.3	**93.5**
9 REPORTING RESULTS		30	1	0	1	93.8	**96.9**
10 LIMITATIONS OF THE SYNTHESIS		29	0	1	1	93.5	**93.5**

4.5 Consensus meeting

An expert panel, comprising members of the project advisory group, plus an additional two methodology experts, participated in a consensus meeting. All participants had expertise in conducting synthesis of quantitative data, including editors of Cochrane Review Groups, and representatives from the Campbell Collaboration and NICE. The group discussed the draft content and wording of each SWiM guideline resulting from the Delphi exercise. Following the meeting, the guideline was finalised and circulated for final comments. In addition, during the finalisation process, we informally piloted the guideline to assess face validity. Eight colleagues, who had not been involved in the Delphi, and with varying levels of experience in conducting systematic reviews, were asked to read and apply the guideline. Short interviews, conducted by MC, were held with the pilot participants to identify any clarification required in the items or their explanations. These colleagues provided feedback on the content and clarity of the guideline items and accompanying explanations.

The feedback was used during the process of finalising the wording of the guideline items and accompanying explanations.

5 Strengths and limitations

Development of the SWiM guideline has followed a best practice approach to developing reporting guidelines. This involved extensive consultation and formal consensus with methodological experts in systematic reviews. The one exception from recommended guideline development practice was that we did not conduct a full comprehensive literature review for methods guidance.[2] Instead, we established there was currently no guideline, and a purposive literature search established the most relevant texts referring to reporting of narrative synthesis, in particular the ESRC guidance.[1] The moderate response rate to the Delphi exercise may be a possible limitation. However, we believe that the expertise of those who took part is more important than the number of participants. All the individuals invited to participate in the Delphi exercise had relevant methodological experience. While we informally piloted the guideline, with colleagues with experience of systematic reviews providing feedback on the content and clarity of the items, more formal assessment of the validity of SWiM would be useful.

Figure S1 Flow chart of review screening

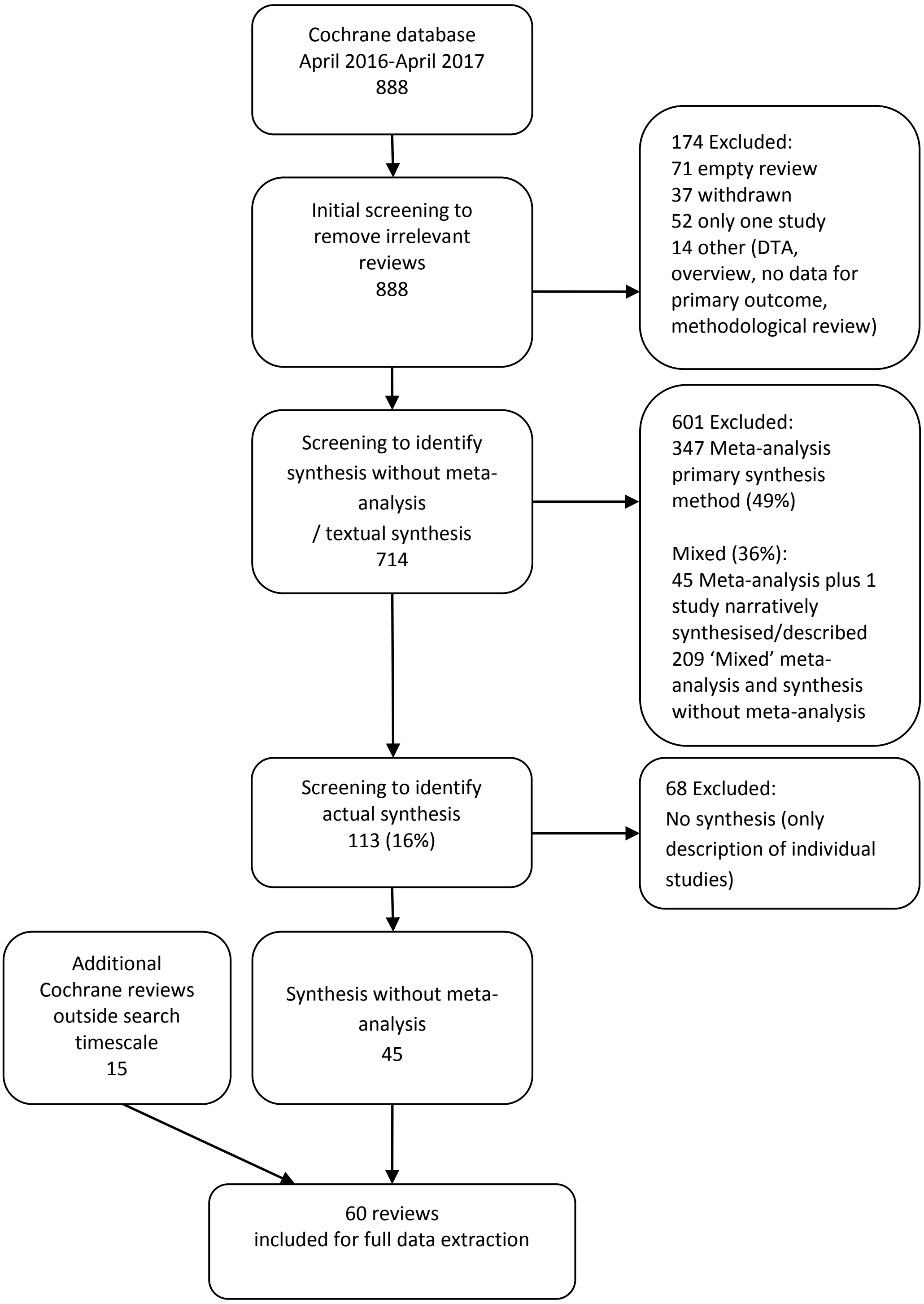

References

1. Popay J, Roberts H, Sowden A, et al. Guidance on the conduct of narrative synthesis in systematic reviews: a product of the ESRC methods programme (Version I) Lancaster, UK: University of Lancaster, 2006.
2. Moher D, Schulz KF, Simera I, et al. Guidance for Developers of Health Research Reporting Guidelines. *PLoS Med* 2010;7(2):e1000217 doi: 10.1371/journal.pmed.1000217.
3. Campbell M, Katikireddi SV, Sowden A, et al. Improving Conduct and Reporting of Narrative Synthesis of Quantitative Data (ICONS-Quant): protocol for a mixed methods study to develop a reporting guideline. *BMJ Open* 2018;8(2) doi: 10.1136/bmjopen-2017-020064.
4. Campbell M, Katikireddi SV, Sowden A, et al. Lack of transparency in reporting narrative synthesis of quantitative data: a methodological assessment of systematic reviews. *Journal of Clinical Epidemiology* 2019;105:1-9
5. Petticrew M, Roberts H. Systematic reviews in the social sciences: a practical guide. *Malden, MA: Blackwell* 2006
6. Armstrong R, Waters E, Jackson N, et al. Guidelines for systematic reviews of health promotion and public health interventions. Version 2. Australia: Melbourne University, 2007.
7. World Health Organization. *WHO Handbook for guideline development*: World Health Organization, 2014.
8. Pope C, Mays N, Popay J. *Synthesising Qualitative and Quantitative Health Evidence: A Guide to Methods*. London: Open University Press, 2007.
9. Gough D, Oliver S, Thomas J. *An introduction to systematic reviews*. London: Sage, 2012.
10. Higgins J, Lasserson T, Chandler J, et al. Methodological Expectations of Cochrane Intervention Reviews. Secondary Methodological Expectations of Cochrane Intervention Reviews 2018. https://community.cochrane.org/mecir-manual/standards-reporting-new-cochrane-intervention-reviews-r1-109/reporting-review-conduct-r1-55/data-collection-analysis-r39-55.
11. Moher D, Liberati A, Tetzlaff J, et al. Preferred reporting items for systematic reviews and meta-analyses: the PRISMA statement. *BMJ* 2009;339(1):b2535- doi: 10.1136/bmj.b2535.
12. Shamseer L, Moher D, Clarke M, et al. Preferred reporting items for systematic review and meta-analysis protocols (PRISMA-P) 2015: elaboration and explanation. *BMJ* 2015;349:g7647 doi: 10.1136/bmj.g7647.
13. Murphy MK, Black NA, Lamping DL, et al. Consensus development methods, and their use in clinical guideline development. *Health Technol Assess* 1998;2(3): 1-88 doi: 10.3310/hta2030.
14. Hoffmann TC, Glasziou PP, Boutron I, et al. Better reporting of interventions: template for intervention description and replication (TIDieR) checklist and guide. *BMJ* 2014;348 doi: 10.1136/bmj.g1687.
15. Beller EM, Glasziou PP, Altman DG, et al. PRISMA for Abstracts: Reporting Systematic Reviews in Journal and Conference Abstracts. *PLoS Med* 2013;10(4):e1001419 doi: 10.1371/journal.pmed.1001419.
