# Supplementary material for: Synthesis without meta-analysis (SWiM) in systematic reviews: reporting guideline
Source: BMJ. 2020 Jan 16;368:l6890. doi: 10.1136/bmj.l6890 (PMC7190266; doi:10.1136/bmj.l6890)
Supplement: Supplementary file 2 — Web appendix: Supplementary file 2 [file camm051291.ww2.pdf]

Synthesis without meta-analysis (SWiM) in systematic reviews: reporting guideline

Supplementary file 2

Table S1 Examples illustrating Synthesis Without Meta-analysis reporting items

**Reporting item**	**Item description with examples**<br>Where possible we have provided published examples. On occasion, we provide clarifying text in square brackets [ ]. References are provided in Table S2 below.
**METHODS**	
**1 Grouping studies for synthesis**	**1a) Provide a description of, and rationale for, the groups used in the synthesis (e.g. groupings of populations, interventions, outcomes, study design)**<br><br>**Review of housing improvement interventions for general, respiratory, and mental health. Example of reporting studies grouped by intervention type and outcome.**<br>*"The studies were grouped by intervention type as follows: improvements in warmth and energy efficiency (after 1985); rehousing or retrofitting with or without wider neighbourhood renewal (after 1995); provision of basic housing needs in developing countries (after 1990); and rehousing from slum conditions (before 1975). …Health impact data were grouped according to 4 categories: general health, respiratory health, mental health, and illness or symptoms" Thomson et al 2009 (page 682)*<br><br>**Review of interventions for enhancing adherence to dietary advice for preventing and managing chronic diseases. Example of reporting studies grouped by intervention, with rationale for groups used.**<br>*"we grouped interventions according to the intervention functions of the behaviour change wheel developed by Michie and colleagues (Michie 2011). Therefore, we classified interventions to enhance adherence to dietary advice as: • Education (increasing knowledge or understanding); • Persuasion (using communication to induce positive or negative feelings or stimulate action); • Incentivisation (creating expectation of reward); • Coercion (creating expectation of punishment or cost); • Training (imparting skills); • Restriction (using rules to reduce the opportunity to engage in the target behaviour); • Environmental restructuring (changing the physical or social context); • Modelling (providing an example for people to aspire to or imitate); • Enablement (increasing means/reducing barriers to increase capability or opportunity); • Multiple (combination of two or more different interventions)." Desroches et al 2013 (page 5)*<br><br>**Review of regional anaesthesia to prevent chronic pain after surgery. Example of reporting studies grouped by intervention, with rationale for groups used.**<br>*"We grouped studies according to surgical interventions (thoracotomy, limb amputation, breast cancer surgery, laparotomy, and other) instead of pooling across different surgical interventions: each surgical intervention has a different natural history of chronic pain.[3]" Andreae et al 2013 (page 712)*
	**1b) Detail and provide rationale for any changes made subsequent to the protocol in the groups used in the synthesis**<br><br>**Review of face-to-face interventions for informing or educating parents about early childhood vaccination. Example of reporting how the mode of intervention delivery was considered in determining the intervention (comparison) groups, with rationale for change from original review to update.**<br>*"This updated review addressed two comparisons:*<br>*1. Face-to-face interventions directed to parents versus control (usual care or passive intervention, i.e. non-face-to-face information or education, or no intervention),*

	2. Face-to-face intervention A versus face-to-face intervention B.<br>*We reduced the comparisons from the original review, which considered the effects of the intervention when directed to individual parents or to groups of parents. There was no clear evidence to suggest that education delivered in a group setting was likely to work differently from education delivered to individuals, and so we felt that this comparison was less informative for end users of the review." Kaufman et al 2018 (page 12)*<br><br>**Review of system change interventions within healthcare settings, for smoking cessation or the provision of smoking cessation care. Example of reporting a change to grouping outcomes between the protocol and the review.**<br>*"We have changed the classification of secondary outcomes, by combining the health professional and patient-level outcomes and reporting them as 'outcome of care'." Thomas et al 2017 (page 41)*
**2 Describe the standardized metric and transformation method used**	**Describe the standardised metric for each outcome. Explain why the metric(s) was chosen, and describe any methods used to transform the intervention effects, as reported in the study, to the standardised metric, citing any methodological guidance used**<br><br>**Review of cognitive behavioural therapy for insomnia and cognitive performance. Example of reporting use of standardized mean difference as the standardized metric.**<br>*"The* SMD [standardized mean difference] *was used as a measure of effect size and was calculated for studies utilising an RCT design, providing the necessary post-treatment and follow-up data. … The SMD was derived by subtracting the mean for the control group from the mean for the treatment group and dividing the result by the pooled standard deviation." Herbert et al 2018 (page 39)*<br><br>**Review of antibiotic therapy in neonates and impact on gut microbiota and antibiotic resistance development. Example of reporting direction of effect standardized metric.**<br>*"We applied a simple vote-counting method to investigate whether the different categories of antibiotic therapy had any effect on the outcomes of interest.[22]* ***Studies were classified based on whether they showed a reduction in the outcome measure, no effect or an increase in the outcome measure following antibiotic treatment.*** *When appropriate, outcomes were presented in vote-count figures." Fjalstad et al 2018 (page 570)*<br><br>**Hypothetical example reporting direction of effect standardized metric.**<br>"*Of the 15 results, only five reported data suitable for meta-analysis (effect estimate and measure of precision (Table 12.5.c, column 8), and no studies reported precise p values.* ***This led the review authors to use vote counting based on direction of effect. For each study, the effect was categorized as beneficial or harmful based on the direction of effect (indicated as a binary metric*** *Table 12.5.c, column 9)." McKenzie and Brennan 2019 (page 345)*<br><br>**Review of homeopathy for any condition. Example of reporting p values standardized metric.**<br>*"The evidence was synthesised by combining* ***the significance levels (P values) for the primary outcomes from the individual trial****s." Cucherat et al 2000 (page 27)*
**3 Describe the synthesis methods**	**Describe and justify the methods used to synthesise the effects for each outcome when it was not possible to undertake a meta-analysis of effect estimates**<br><br>**Review of homeopathy for any condition. Example of reporting combining p values synthesis method.**<br>*"The statistical approach used, therefore, was the combination of the significance levels*

	(P values)[6-8]. The rationale for this choice is that all the trials explored the same broad question, i.e. 'is homeopathic treatment efficacious?', even if, for individual trials, the question asked expressed more specific terms and focused on a given treatment of a particular disease. … Using this approach, the null hypothesis tested is that the effect of interest (in this case, the efficacy of homeopathic treatment) is not present in any of the trials considered. If the null hypothesis is rejected, we can conclude that in at least one trial there is a non-null effect. … [further explanation provided of method] … The major advantage of this approach is that P values from any statistical test for the hypothesis of interest can be combined. If the results are interpreted with sufficient precaution, this approach provides a way to combine results from very dis-similar trials with differing outcomes and statistical tests. … Thus, we used seven methods: the sum of logs, the sum of Z, the weighted sum of Z, the sum of t, the mean Z, the mean P, the count test and the logit procedure." Cucherat et al 2000 (page 28)<br><br>**Review of reminders to healthcare professionals on quality of care, computer-generated or delivered on paper, on healthcare professionals' practice outcomes and patient health outcomes. Example of reporting summary of effect estimates synthesis method.**<br>*"We combined cluster- and patient-randomized trials using the median improvement and interquartile range (IQR). … Briefly, each study is represented by a single representative outcome and the median effect size and IQR are calculated across the included studies. By using the median rather than the mean, the summary estimate is less likely to be influenced by outlying results (e.g. large effects from methodologically poor studies)." Arditi et al 2017 (page 10)*<br><br>**Review of mass media interventions for reducing mental health-related stigma. Example of reporting vote counting by direction of effect synthesis and summary of effect estimates methods.**<br>*"… for each comparison (e.g. mass media intervention versus control) we stated: the number of comparisons showing a positive direction of effect; the median effect size across all comparisons; the median effect size across comparisons without unit of analysis errors; and the number of comparisons showing statistically significant effects. This approach was recommended by Grimshaw 2003 as it 'allows the reader to assess the likely effect size and consistency of effects across all included studies and whether these effects differ between studies, with and without unit of analysis errors'." Clement et al 2013 (page 13)*<br><br>**Review of skin status to predict ulcer development. Example of reporting vote counting by direction of effect synthesis method.**<br>*"For studies without data for a meta-analysis of effect sizes, we used the sign test to judge the existence of prognostic association (Borenstein et al., 2009). In sign tests, we counted the number of studies with associations in one direction (i.e., point estimate falls in one side of non-effect) and compared this with the number of studies with associations in another direction (Borenstein et al., 2009)." Shi et al 2018 (page 16)*
**4 Criteria used to prioritise results for summary and synthesis**	**Where applicable, provide the criteria used, with supporting justification, to select particular studies, or a particular study, for the main synthesis or to draw conclusions from the synthesis (e.g. based on study design, risk of bias assessments, directness in relation to the review question)**<br><br>**Review of housing improvement interventions for general, respiratory, and mental health. Example of reporting studies prioritized by study quality.**<br>*"… a synthesis of the reported impacts for the better quality experimental and non-experimental studies (Overall Grade A and B) was presented at the start of each outcome domain. … The findings of those studies assessed to have an Overall Grade of C were reported but not included in the final synthesis. The poorer quality studies (Overall Grade C) were examined to identify additional impact types reported and the existence*

	of adverse impacts not reported in other studies." Thomson et al 2013 (page 30)<br><br>**Review of interventions to enhance medication adherence. Example of reporting studies prioritized by risk of bias.**<br>*"To provide a reasonable sampling of the details and diversity of included adherence intervention RCTs, we chose to provide a narrative focused strictly on the RCTs with the lowest risk of bias. … we conducted a narrative analysis of the studies with the lowest risk of bias, using the Cochrane 'Risk of bias' tool (Higgins 2011), for study design (random sequence generation and concealment of allocation), and for their primary clinical outcome (blinding of outcome assessor, as relevant for the outcome in question). We did not require low risk of bias for the primary adherence measure, as this would have resulted in discussion of very few studies, as most adherence measures have a high or uncertain risk of bias, notably because of lack of blinding. … We reported effects in the RCTs identified as low risk of bias as described by the authors, with a focus on the primary outcomes, and with comments on methodological issues that might have influenced results, such as absence of correction for multiple comparisons." Neiuwlaat et al 2014 (page 9)*
**5 Investigation of heterogeneity in reported effects**	**State the method(s) used to examine heterogeneity in reported effects when it is not possible to undertake a meta-analysis of effect estimates and its extensions to investigate heterogeneity**<br><br>**Review of slum upgrading interventions and health. Example of reporting exploration of heterogeneity using harvest plots**<br>*"harvest plots were developed to visually compare results between single and multicomponent interventions and those with and without community involvement." Turley et al 2013 (page 42)*<br><br>**Review of system change interventions for smoking cessation. Example of reporting exploration of heterogeneity using tables and forest plots.**<br>*"We explored heterogeneity visually using tables and forest plots, by comparing the effect sizes of studies grouped according to potential effect modifiers. These included: Type of intervention (e.g. identification of smokers, documentation of smoking status, treatment, training of health professionals, feedback of services, etc.); Intensity of intervention (e.g. counselling, pharmacotherapy, both counselling and pharmacotherapy, duration of intervention, etc.); Type of health professional involved; Setting (primary, secondary and tertiary); Study design (RCTs, cluster-RCTs, quasi-RCTs or ITS studies); Quality of studies." Thomas et al 2017 (page 8)*<br><br>**Review of interventions for improving the appropriate use of imaging in people with musculoskeletal conditions. Example of reporting exploration of heterogeneity using table of median effect sizes.**<br>*We performed exploratory analyses to determine whether different study characteristics varied the effects of the interventions. The results of these analyses are presented in Table 8* [reports the median and range of the observed effects (adjusted risk differences)]. *Some studies targeted a decreased use of imaging (for example, decreased number of lumbar x-rays ordered) and other studies targeted an increased use of imaging (for example, increased use of BMD test ordering). We examined whether this different type of targeted behaviour modified, on average, the effect of the interventions." French et al 2010 (page 14)*
**6 Certainty of evidence**	**Describe the methods used to assess certainty of the synthesis findings**

	**Review of mechanical versus manual chest compressions for cardiac arrest. Example of reporting methods for assessing certainty.**<br>*"In this 2017 update, in line with changes in the MECIR standards, we explicitly evaluated the quality* [certainty] *of evidence for each outcome using the GRADE approach (Ryan 2016). Given that all outcomes were from RCTs, the starting rating of evidence was 'high quality'* [certainty]. *We downgraded quality* [certainty] *by one level for serious concerns and two levels for very serious concerns regarding risk of bias, inconsistency, indirectness, imprecision, and publication bias." Wang et al 2018 (page 10)*<br><br>**Review of interventions to improve adherence to Standard Precautions for the control of health care-associated infections. Example of reporting methods for assessing certainty.**<br>*"We summarised the findings for each intervention strategy using the GRADE approach. Two review authors (DM and RED) independently assessed the certainty of evidence (high, moderate, low, and very low) using the five GRADE considerations (risk of bias, consistency of effect, imprecision, indirectness, and publication bias) for each of the following outcomes to draw conclusions about certainty of the evidence: adherence to Standard Precautions; healthcare workers' knowledge; and rates of health care-associated colonisation with MRSA* [Methicillin-resistant Staphylococcus aureus] *(Guyatt 2008). We used methods and recommendations described in Section 8.5 and Chapter 12 of the Cochrane Handbook for Systematic Reviews of interventions and EPOC worksheets (EPOC 2013; Higgins 2011). We resolved disagreements on certainty ratings by discussion and provided justification for decisions to downgrade or upgrade ratings using table footnotes." Moralejo et al 2018 (page 10)*
**7 Data presentation methods**	**Describe the graphical and tabular methods used to present the effects (e.g. tables, forest plots, harvest plots)**<br>**Specify key study characteristics (e.g. study design, risk of bias) used to order the studies, in the text and any tables or graphs, clearly referencing the studies included**<br><br>**Review of slum upgrading interventions and health. Example of reporting data presentation using harvest plots.**<br>*"Drawing on methods described by Ogilvie 2008, harvest plots were developed to visually convey findings, appropriateness of the study design, confidence in the estimate of effect and risk of bias of the included studies. Studies are represented by bars plotted onto a grid, marked with the first three letters of the primary author's surname and placed according to the direction of effect. Outcome measures varied considerably between studies, thus study results were grouped into broad outcome categories. For example, diarrhoea, parasitic infections and dengue fever were all plotted within the communicable diseases group. Where possible, one bar was used for each study within one outcome group. In some cases, the same study measured two or more individual outcomes that fitted under the same broad outcome heading. Where the direction and statistical significance of the effects were the same, one bar was used for all outcome indicators. Where a study's results differed for each indicator, separate bars were used to illustrate the uncertainty." Turley et al 2013 (page 15)*<br><br>**Review of housing improvement interventions for general, respiratory and mental health. Example of reporting data presentation using tables.**<br>*"Many of the trials reported several measures of the same outcome and/or measured outcomes at different time points, resulting in multiple comparisons for each outcome. The findings are summarised by outcome in table 3 and are described below. … An effect direction plot provides a visual display of the results across all outcome domains, ordered by risk of bias and the intensity of the intervention (table 4)."* Hurt et al 2018 (page 5)

	**Review of housing improvement interventions for general, respiratory, and mental health. Example of reporting data presentation using an effect direct plot.**<br>*"A summary of available standardized effect estimates is presented in the Data and analyses section (see also Figure 5; Figure 6; Figure 7; Table 12). In addition, a visual summary of the direction of all reported impacts has also been tabulated (Table 10) in what we call an 'effect direction plot'. The effect direction plot allows for effect directions of multiple outcomes and intermediate outcomes, such as change in housing conditions, to be summarized visually. The plot included an indication of study design, study quality, study size, as well as the type of analysis presented in each study or where no statistics were available …* [table heading] *Summary of included study characteristics and findings (ordered by study quality (Hamilton Overall Grade), date of publication and study design)." Thomson et al 2013 (page 28)*<br><br>**Review of interventions to improve imaging for musculoskeletal conditions. Example of specifying study characteristics used to order the studies.**<br>*"We reported the synthesis of results of the included studies classified by condition (osteoporosis, low back pain, knee pain, other musculoskeletal conditions) in the following structured format: 1. Evaluations of interventions against 'no-intervention' control groups. 2. Evaluations of interventions against 'other intervention' control groups" French et al 2010 (page 5)*<br><br>**Review of local treatments for metastases of renal cell carcinoma. Example of reporting data presentation using forest plots.**<br>*"When pooling of data was not done, and where appropriate, results were presented in forest plots to allow a visual comparison of the effects of interventions between studies." Dabestani et al 2014 (page 550)*
**RESULTS**	
**8 Reporting results**	**For each comparison and outcome, provide a description of the synthesised findings, and the certainty of the findings. Describe the result in language that is consistent with the question the synthesis addresses, and indicate which studies contribute to the synthesis**<br><br>**Review of reminders (computer-generated or on paper) for healthcare professionals on the quality of care, on healthcare professionals' practice outcomes and patient health outcomes. Example of reporting summary statistics of observed effect estimates and certainty of evidence.**<br>*"Computer-generated reminders delivered on paper to healthcare professionals, alone (single-component intervention) or in addition to co-intervention(s) (multi-component intervention), probably improve slightly quality of care compared with usual care or the co-intervention(s) without the reminder component (median improvement 6.8% (IQR: 3.8% to 17.5%); 34 studies (40 comparisons); moderate-certainty evidence) (see Summary of findings for the main comparison)." Arditi et al 2017 (page 18)*<br><br>**Hypothetical example. Review of midwife-led continuity models of care versus other models of care. Example of reporting synthesis of p values for maternal satisfaction of care.**<br>*"There was strong evidence of benefit of midwife-led models of care in at least one study (P value < 0.001, 13 studies). However, a sensitivity analysis restricted to studies with an overall low risk of bias suggested there was no effect of midwife-led models of care in any of the trials (P value = 0.314, 3 studies). Estimated standardized mean differences for five of the outcomes were small (ranging from -0.13 to 0.45) (Figure 12.4.b, Panel C)." McKenzie and Brennan 2019 (page 344)*<br><br>**Hypothetical example. Review of midwife-led continuity models of care versus other models of care. Example of reporting synthesis using vote counting based on direction of effects for maternal satisfaction of care.**

	"There was evidence that midwife-led models of care had an effect on satisfaction, with 10 of 12 studies favouring the intervention (83% (95%CI 55% to 95%), P value = 0.039) (Figure 12.4.b, Panel D). Four of the 12 studies were judged to be at low risk of bias [6,9,14,15], *and three of these favoured the intervention* [6,14,15]. *The available effect estimates are presented in…Table X." McKenzie and Brennan 2019 (page 345)*
**Discussion**	
**9 Limitations of the synthesis**	**Report the limitations of the synthesis methods used and/or the groupings used in the synthesis, and how these affect the conclusions that can be drawn in relation to the original review question**<br><br>**Review of reminders to healthcare professionals on quality of care, computer-generated or delivered on paper, on healthcare professionals' practice outcomes and patient health outcomes. Example of reporting limitation of the metric and synthesis method used.**<br>*"Using the median effect size as analytic approach allowed us to avoid the unit of analysis issues in unadjusted cluster trials, but it limits the interpretability of the results as there are no confidence intervals of individual effect sizes indicating the degree of imprecision of the results." Arditi et al 2017 (page 27)*<br><br>**Review of antibiotic therapy in neonates and impact on gut microbiota and antibiotic resistance development. Example of reporting limitation of synthesis method used.**<br>*"The main limitations were the lack of RCTs and the diverse study outcomes, which made meta-analysis impossible to perform. Instead, we applied a … vote-counting method to assess the effect of neonatal antibiotic treatment on relevant outcomes. This method has limitations as it usually fails to account for the population size. … Nevertheless, vote counting may be an effective method to assess the ranking of outcomes.[100]" Fjalstad et al 2018 (page 576)*<br><br>**Review of health benefits of green spaces in the living environment. Example of limitation of grouping outcomes at a higher level, which precluded synthesis of specific green space quantity measures.**<br>*"Another limitation is that to enable an evidence synthesis, two types of green space quantity measures were combined. When more studies become available, it will be possible to investigate whether relationships with health outcomes differ for different green space quantity measures." Van den Berg et al 2015 (page 814)*

Table S2 References for guideline examples

Item	Citation
1a	**Thomson** H, Thomas S, Sellstrom E, Petticrew M. The Health Impacts of Housing Improvement: A Systematic Review of Intervention Studies From 1887 to 2007. *Am J Public Health* 2009;99(S3): S681-S692. (page 682)<br><br>**Desroches** S, Lapointe A, Ratté S, et al. Interventions to enhance adherence to dietary advice for preventing andmanaging chronic diseases in adults. *Cochrane Database Syst Rev* 2013(2):CD008722. (page 5)<br><br>**Andreae** M, Andreae D. Regional anaesthesia to prevent chronic pain after surgery: a Cochrane systematic review and meta-analysis. *Br J Anaesth* 2013;111(5):711-20 (page 712)
1b	**Kaufman** J, Ryan R, Walsh L, Horey D, Leask J, Robinson P, Hill S. Face-to-face interventions for informing or educating parents about early childhood vaccination. *Cochrane Database Syst Rev* 2018;(5):CD010038. (page 12)<br><br>**Thomas** D, Abramson MJ, Bonevski B, George J. System change interventions for smoking cessation. *Cochrane Database Syst Rev* 2017;(2):CD010742. (page 41)
2	**Herbert** V, Kyle SD, Pratt D. Does cognitive behavioural therapy for insomnia improve cognitive performance? A systematic review and narrative synthesis. *Sleep Med Rev* 2018;39:37-51 (page 39)<br><br>**Fjalstad** JW, Esaiassen E, Juvet LK, et al. Antibiotic therapy in neonates and impact on gut microbiota and antibiotic resistance development: a systematic review. *J Antimicrob Chemother* 2017;73(3):569-80 (page 570)<br><br>**McKenzie** J, Brennan S. Synthesizing and presenting findings using other methods. In: Higgins J, Thomas J, Chandler J, et al, eds. *Cochrane Handbook for Systematic Reviews of Interventions.* 2nd ed. John Wiley & Sons, 2019: 321-48 (page 345)<br><br>**Cucherat** M, Haugh MC, Gooch M, Boissel JP. (2000). Evidence of clinical efficacy of homeopathy. *Eur J Clin Pharmacol* 2000;56(1): 27-33. (page 27)
3	**Cucherat** M, Haugh MC, Gooch M, Boissel JP. (2000). Evidence of clinical efficacy of homeopathy. *Eur J Clin Pharmacol* 2000;56(1): 27-33. (page 28)<br><br>**Arditi** C, Rège-Walther M, Durieux P, Burnand B. Computer-generated reminders delivered on paper to healthcare professionals: effects on professional practice and healthcare outcomes. *Cochrane Database Syst Rev* 2017(7):CD001175. (page 10)<br><br>**Clement** S, Lassman F, Barley E, et al. Mass media interventions for reducing mental health-related stigma. *Cochrane Database Syst Rev* 2013(7):CD009453. (page 13)<br><br>**Shi** C, Dumville JC, Cullum N. Skin status for predicting pressure ulcer development: a systematic review and meta-analyses. *Int J Nurs Stud* 2018;87:14-25 (page 16)
4	**Thomson** H, Thomas S, Sellstrom E, Petticrew M. Housing improvements for health and associated socio-economic outcomes. *Cochrane Database Syst Rev* 2013;(2):CD008657. (page 30)<br><br>**Nieuwlaat** R, Wilczynski N, Navarro T, et al. Interventions for enhancing medication adherence. *Cochrane Database Syst Rev* 2014(11):CD000011. (page 9)
5	**Turley** R, Saith R, Bhan N, Rehfuess E, Carter B. Slum upgrading strategies involving physical environment and infrastructure interventions and their effects on health and socio-economic

	outcomes. *Cochrane Database Syst Rev* 2013;(1):CD010067. (page 42)<br><br>**Thomas** D, Abramson MJ, Bonevski B, George J. System change interventions for smoking cessation. *Cochrane Database Syst Rev* 2017;(2):CD010742. (page 8)<br><br>**French** SD, Green S, Buchbinder R, et al. Interventions for improving the appropriate use of imaging in people with musculoskeletal conditions. *Cochrane Database Syst Rev* 2010(1):CD006094. (page 14)
6	**Wang** P, Brooks S. Mechanical versus manual chest compressions for cardiac arrest. *Cochrane Database Syst Rev* 2018(8):CD007260. (page 10)<br><br>**Moralejo** D, El Dib R, Prata RA, et al. Improving adherence to Standard Precautions for the control of health care-associated infections. *Cochrane Database Syst Rev* 2018(2):CD010768. (page 10)
7	**Turley** R, Saith R, Bhan N, Rehfuess E, Carter B. Slum upgrading strategies involving physical environment and infrastructure interventions and their effects on health and socio-economic outcomes. *Cochrane Database Syst Rev* 2013;(1):CD010067. (page 15)<br><br>**Hurt** L, Paranjothy S, Lucas PJ, et al. Interventions that enhance health services for parents and infants to improve child development and social and emotional well-being in high-income countries: a systematic review. *BMJ Open* 8(2): e014899. (page 5)<br><br>**Thomson** H, Thomas S, Sellstrom E, et al. Housing improvements for health and associated socio-economic outcomes. *Cochrane Database Syst Rev* 2013(2):CD008657. (page 28)<br><br>**French** SD, Green S, Buchbinder R, et al. Interventions for improving the appropriate use of imaging in people with musculoskeletal conditions. *Cochrane Database Syst Rev* 2010(1):CD006094. (page 5)<br><br>**Dabestani** S, Marconi L, Hofmann F, et al. Local treatments for metastases of renal cell carcinoma: a systematic review. *Lancet Oncol* 2014;15(12):e549-e61. (page 550)
8	**Arditi** C, Rège-Walther M, Durieux P, Burnand B. Computer-generated reminders delivered on paper to healthcare professionals: effects on professional practice and healthcare outcomes. *Cochrane Database Syst Rev* 2017(7):CD001175. (page 18)<br><br>**McKenzie** J, Brennan S. Synthesizing and presenting findings using other methods. In: Higgins J, Thomas J, Chandler J, et al, eds. *Cochrane Handbook for Systematic Reviews of Interventions.* 2nd ed. John Wiley & Sons, 2019: 321-48 (page 344 and 345)
9	**Arditi** C, Rège-Walther M, Durieux P, Burnand B. Computer-generated reminders delivered on paper to healthcare professionals: effects on professional practice and healthcare outcomes. *Cochrane Database Syst Rev* 2017(7):CD001175 (page 27)<br><br>**Fjalstad** JW, Esaiassen E, Juvet LK, et al. Antibiotic therapy in neonates and impact on gut microbiota and antibiotic resistance development: a systematic review. *J Antimicrob Chemother* 2017;73(3):569-80 (page 570)<br><br>**van den Berg** M, Wendel-Vos W, van Poppel M, Kemper H, W. van Mechelen W, and Maas J. (2015). Health benefits of green spaces in the living environment: a systematic review of epidemiological studies. *Urban For Urban Gree* 2015 14(4): 806-816 (page 814)
